# SARS-CoV-2 Omicron variant: viral spread dynamics, disease burden, and vaccine effectiveness

**DOI:** 10.1007/s44194-022-00014-x

**Published:** 2022-08-30

**Authors:** Shengzhi Sun, Jiong Wu, Rui Chen, Michael Levitt

**Affiliations:** 1 School of Public Health, Capital Medical University, Beijing, 100069 China; 2 School of Life Sciences, Jiangsu Normal University, Xuzhou, 221116 China; 3 Department of Structural Biology, Stanford School of Medicine, Stanford, CA 94305 USA

**Keywords:** Omicron, SARS-CoV-2, spread dynamics, Gompertz growth function

The first coronavirus disease 2019 (COVID-19) case, caused by the severe acute respiratory syndrome coronavirus 2 (SARS-CoV-2) virus, was reported by officials in December 2019 in Wuhan, China. The COVID-19 has since spread worldwide, leading to the ongoing pandemic.

There have been a few waves of infection caused by different COVID-19 variants of concern. The Omicron (B.1.1.529), the newly emerged variant of concern, has fueled a surge in daily cases in recent months than any previous variants. To inform response measures to COVID-19, we provide what we learned from the pandemic caused by the Omicron variant.

## The dynamics of the Omicron variant spread

**Fig. 1:**
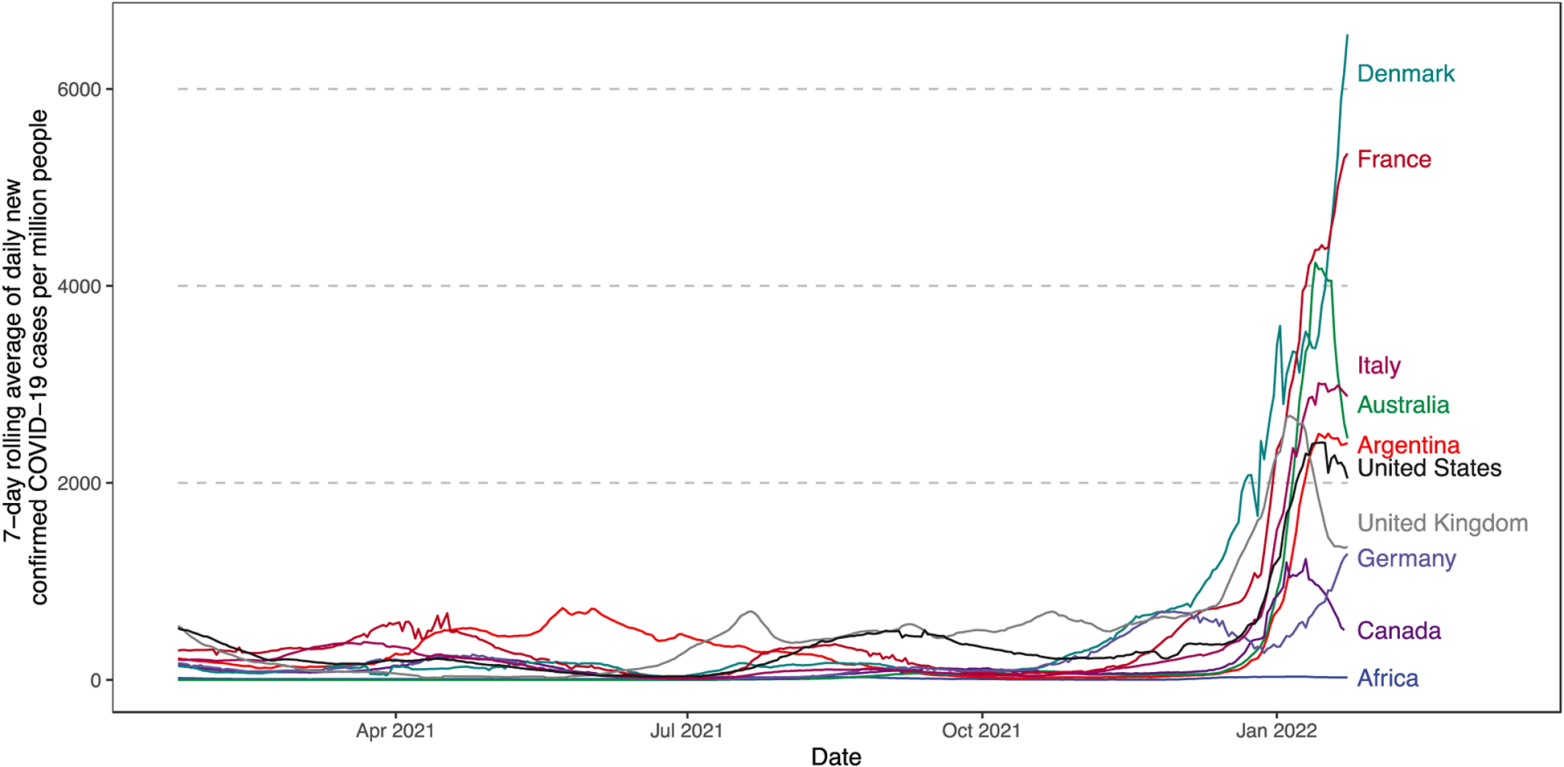
Seven-day rolling average of daily new confirmed COVID-19 cases per million people in selected countries between January 23, 2021, and January 24, 2022

The Omicron variant is more transmissible than any previous variants of concern (Accorsi et al. 2022; Andrews et al. 2022). Early studies suggested that the Omicron variant was 2.6 to 3.7 times more contagious than the Delta variant among vaccinated people (Accorsi et al. 2022). This is also supported by an alarming spike in daily confirmed cases in late January2022 driven by the Omicron variant, which broke records in most countries in the world (Fig. 1).

**Fig. 2:**
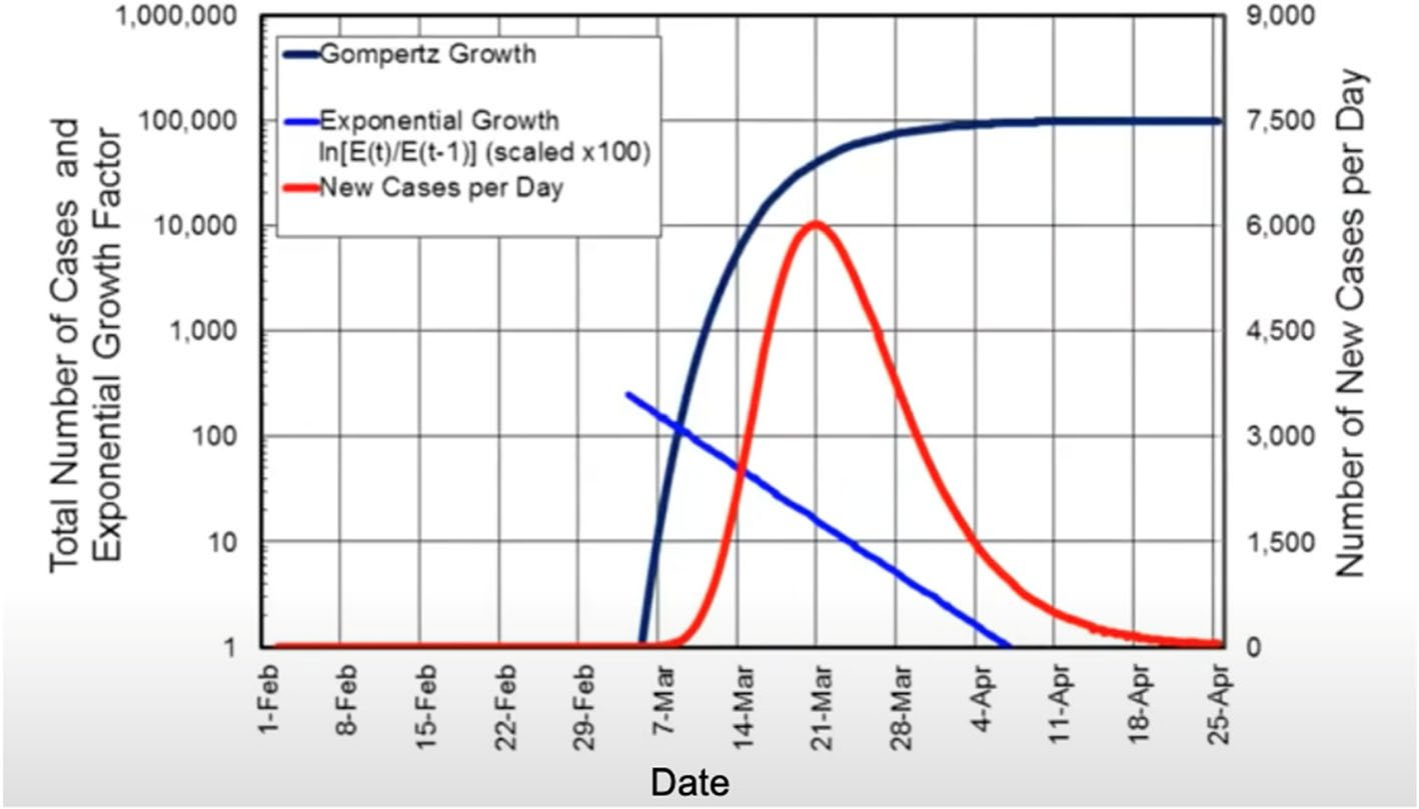
Properties of the Gompertz growth function

**Fig. 3:**
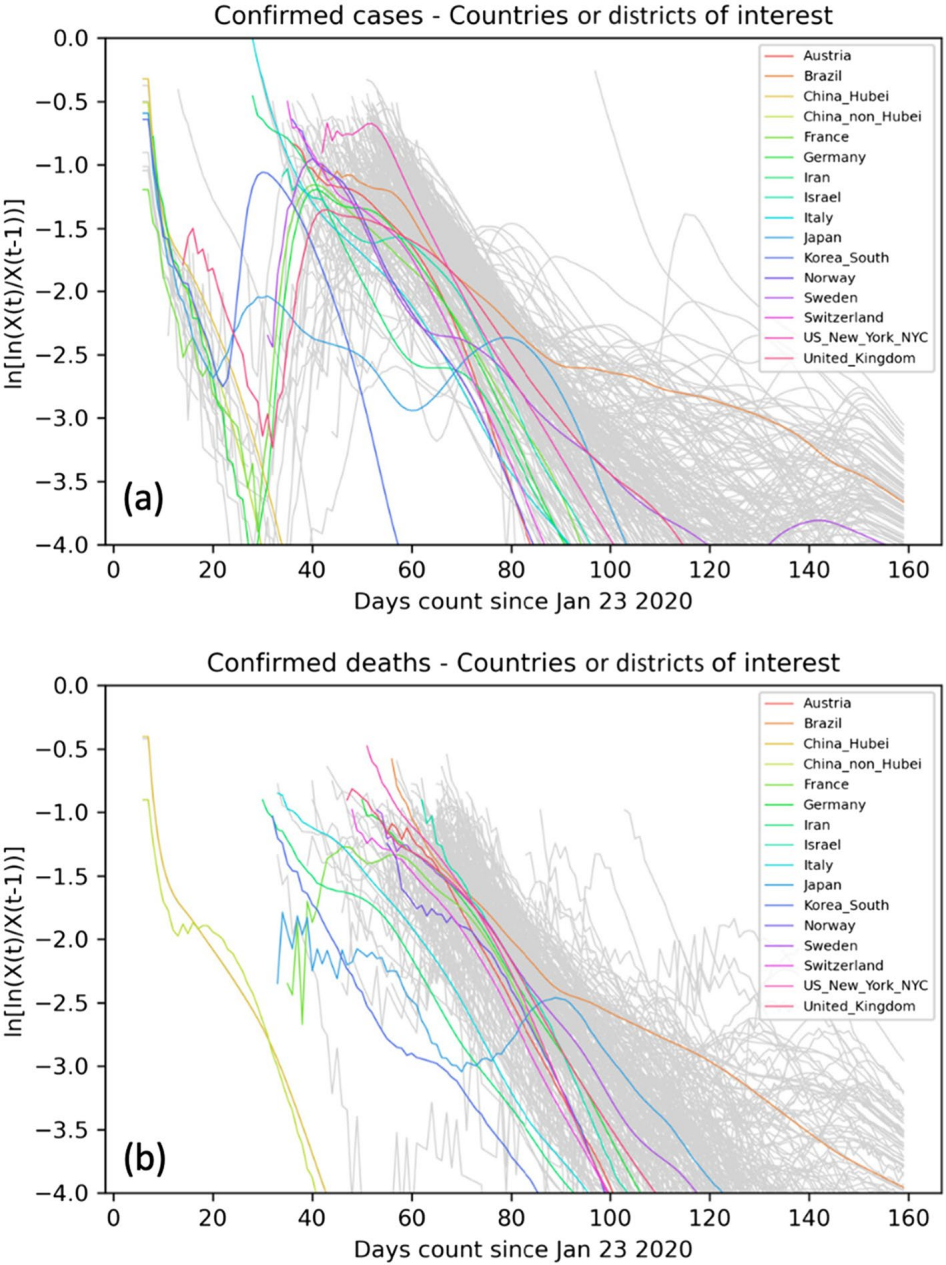
The exponential growth of COVID-19 cases and deaths decreases linearly as expected for the Gompertz growth function by countries or districts in 2020. Source: adapted from Levitt et al. (2020) (Buchan et al. 2022).

**Fig. 4:**
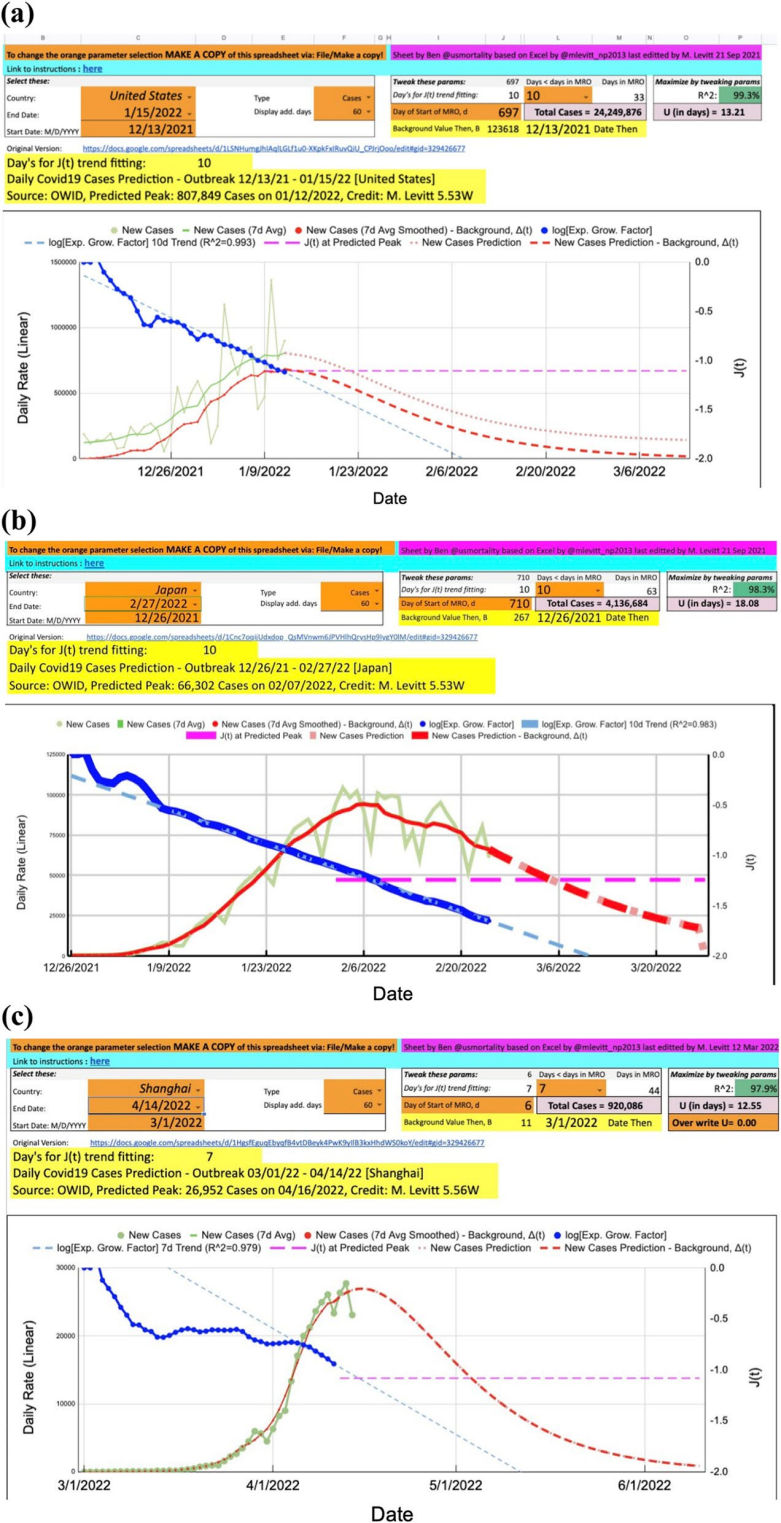
The Gompertz growth curve of COVID-19 cases in (**a**) the United States, (**b**) Japan, and (**c**) Shanghai, China in 2022

A comprehensive analysis of SARS-CoV-2 individual clearly resolved outbreaks in 113 world locations shows that the growth of a COVID-19 pandemic follows a Gompertz growth function (Buchan et al. 2022). Like the outbreaks caused by previous COVID-19 variants of concern, the count of confirmed cases and deaths caused by the Omicron variant also follows the Gompertz growth function and is predictable and self-limiting (Fig.2) (Buchan et al. 2022; Cai et al. 2022). In addition, the trajectory of the COVID-19 pandemic is universally consistent across countries with differences in social structures and preventive and treatment measures responding to the virus (Figs. 3 and 4). Because of this property of the COVID-19 pandemic, the entire trajectory can be predicted early, which might be used to guide preparation and resources allocation.

## Disease burden of Omicron variant

The illness caused by the Omicron variant is generally milder than that associated with SARS-CoV-2 wild type and other variants of concern. In an analysis of 161,328 individuals with COVID-19 diagnosed between October 1, 2021 and December 6, 2021in South Africa, the odds of hospital admission for patients infected with the Omicron variant was 80% (95% CI: 70%, 90%) lower than other SARS-CoV-2 variants during the same period, and the odds of severe disease was 70% (95% CI: 50%, 80%) lower among patients infected with the Omicron variant versus the Delta variant in earlier epidemic waves (Cheung et al. 2022). A non-peer-reviewed report of 52,297 Omicron cases and 16,982 Delta cases in the United States (US) reported that the infection of the Omicron variant was associated with a 53% (95% CI: 38%, 65%) lower risk of symptomatic hospitalization, 74% (95% CI: 27%, 90%) lower risk of intensive care unit admission, 91% (95% CI: 25%, 99%) lower risk of death, and 52% (95% CI: 36%, 64%) lower risk of any subsequent hospitalization as compared to the infection of the Delta variant (UK Health Security Agency 2021). The reduced risk of hospitalization for the Omicron variant was also reported in many other countries, such as the United Kingdom (Ferguson et al. 2022), and Canada (Garret et al. 2021).

In terms of the death burden of the Omicron variant, we compared the death rate between SARS-CoV-2 wild type and influenza in the US. The Diamond Princess Cruise ship can be seen as an unintended experiment to infect all the passengers with the SARS-CoV-2 wild type, representing the worst-case scenario in regard to disease spread. Approximately 1,690 people on the Diamond Princess were over 65 years old, and only 20% of people were infected, and the death rate was 0.41%. By comparison, from 2017 to 2018, the death rate for the influenza population was 0.096% in the US (Levitt et al. 2020). This means that if COVID-19 spreads everywhere as influenza has, it will be 4.3 times more lethal than influenza was to people in the US over 65 years of age from 2017 to 2018. If the older people on the cruise were particularly unhealthy or the illness caused by the Omicron variant was milder than that associated with SARS-CoV-2 wild type, the difference in death rate between Omicron variant and influenza would be much less.

In short, if the infection is allowed to run its course and infects a large fraction of the population, the expected death toll will be 2 to 4 times higher than that of the worst influenza in the past decade; in other words, the additional deaths would be equal to approximately 1 to 2 months of natural deaths each year, with the majority of deaths occurring among the elderly.

## COVID-19 vaccines are effective against severe conditions

The evidence from epidemiologic studies regarding the effectiveness of vaccines against the Omicron variant is accumulating (Lewnard et al. 2022; Lyngse et al. 2021; Thompson 2022). Emerging evidence suggests that people who received the COVID-19 vaccine have a substantially reduced risk of severe illness and death from the Omicron variant than unvaccinated people (Lewnard et al. 2022; Lyngse et al. 2021; Thompson 2022). For example, in a US multistate analysis of 222,772 emergency room and urgent care encounters and 87,904 hospitalizations among adults with COVID-19 like illnesses between August 26,2021 and January 5, 2022, Thompson et al. (2022) found that the effectiveness of preventing emergency room and urgent care six months after two mRNA vaccine doses for Omicron variant was 38%, and after a third mRNA vaccine, the protection was up to 82%. For hospitalization, vaccine effectiveness of two-dose protection for Omicron infection was 57%, and vaccine effectiveness of three-dose protection was 90% (Lyngse et al. 2021). In Hong Kong, the death rate during the fifth wave of COVID-19 was 0.4% among unvaccinated individuals, 0.09% among individuals having first dose vaccination, and 0.01% among individuals having second/ booster dose COVID-19 vaccination (Levitt et al. 2020).

Taken together, although the Omicron variant is more contagious than any previous variants of concern, the trajectory of the pandemic is predictable and self-limiting. Moreover, even if the Omicron variant is allowed to spread out of control, the death toll will be ~ 4 times higher than that of the worst influenza in the past decade.

## Looking ahead

The SARS-CoV-2 is constantly evolving by acquiring mutations over time. The emerging Omicron sub-lineage BA.2 is the most transmissible among all existing strains of SARS-CoV-2, driving the rising in daily cases in some countries. The Omicron variant has a much higher rate of silent SARS-CoV-2 carriers than the earlier variants (Wolter et al. 2022). These silent carriers are contagious and contribute to the rapid spread of SARS-CoV-2 in the community. It is now widely believed that non-pharmaceutical interventions (e.g., mask-wearing, enhanced testing, social distancing, and reducing mass gatherings) alone might not be sufficient to prevent the outbreak caused by the Omicron variant (Zonta et al. 2021).

What we have learned from the Omicron variant in terms of the dynamics of viral spread, the death burden, and the vaccine effectiveness might be valuable for regions that may anticipate the outbreaks caused by the Omicron variant.
